# Complications Following Primary Repair of Non-proximal Hypospadias in Children: A Systematic Review and Meta-Analysis

**DOI:** 10.3389/fped.2020.579364

**Published:** 2020-12-09

**Authors:** Yuhao Wu, Junke Wang, Tianxin Zhao, Yuexin Wei, Lindong Han, Xing Liu, Tao Lin, Guanghui Wei, Shengde Wu

**Affiliations:** ^1^Department of Urology, Children's Hospital of Chongqing Medical University, Chongqing, China; ^2^Chongqing Key Laboratory of Pediatrics, Chongqing Key Laboratory of Children Urogenital Development and Tissue Engineering, Ministry of Education Key Laboratory of Child Development and Disorders, China International Science and Technology Cooperation Base of Child Development and Critical Disorders, National Clinical Research Center for Child Health and Disorders, Chongqing, China

**Keywords:** surgery, meta-analysis, complications, children, hypospadias

## Abstract

**Purpose:** The aim of this study was to systematically review the literature on the complications and postoperative outcomes of children with non-proximal hypospadias.

**Methods:** Electronic databases including PubMed, Embase, and Cochrane Library CENTRAL were searched systematically from January 1990 to June 2020 for the literature that reported the postoperative outcomes of patients with non-proximal hypospadias. Non-proximal hypospadias encompassed distal and mid-penile hypospadias.

**Results:** We included 44 studies involving 10,666 subjects. Urethrocutaneous fistula (UCF) was the most common complication with an incidence of 4.0% (95% CI, 3.1–5.0%). Incidence of overall complications was 8.0% (95% CI, 6.3–9.8%). Meta-regression analysis revealed that length of urethral stent indwelling (coefficient 0.006; 95% CI, 0.000–0.011; *p* = 0.036) and penile dressing (coefficient 0.010; 95% CI, 0.000–0.021; *p* = 0.048) were two risk factors for UCF. Multivariate meta-regression analysis did not identify any independent risk factors for UCF. No differences were found between stent and stentless groups in non-proximal hypospadias regarding incidences of UCF (OR, 0.589; 95% CI, 0.267–1.297), meatal stenosis (OR, 0.880; 95% CI, 0.318–2.437), and overall complications (OR, 0.695; 95% CI, 0.403–1.199). No differences were found between foreskin preservation and circumcision in terms of complications either.

**Conclusions:** UCF is the most common complication following hypospadias repair with an incidence of 4.0%. Independent risk factors for UCF were not identified in the current research. Distal hypospadias repair without stent indwelling is not likely to compromise the postoperative outcome. Further studies should be designed to explore the differences between different surgical approaches and the potential risk factors for complications following hypospadias repair.

## Introduction

Hypospadias is one of the most common urogenital anomalies in male with an incidence of 1 in 300 live births (1). The condition is typically defined as proximal location of meatus, chordee, and a ventrally deficient foreskin. Repair of hypospadias aims to create a straight penis, a neourethra with a meatus at the tip of glans, and a normal appearance of circumcised phallus. Despite the improvement and evolution in hypospadias repair, no technique has been adopted as the golden standard because no conclusive evidences have shown the superiority of one technique over another (2). Urethrocutaneous fistula (UCF) is the most common complications following hypospadias repair (3).

Proximal hypospadias, encompassing penoscrotal, scrotal, and perineal hypospadias, still remains the most challenging conditions for pediatric urologists. Several surgical strategies (i.e., transverse preputial island flap, also known as Duckett repair) are designed for proximal hypospadias; however, complications such as UCF and urethral diverticulum are common following proximal hypospadias repair (4).

As compared with proximal condition, non-proximal hypospadias, which encompasses distal and mid-penile hypospadias, is a quite different entity with a much higher incidence. In current literature, incidence of complications varies significantly since highly divergent surgical approaches have been introduced for non-proximal hypospadias repair, and the exact incidence of complications following hypospadias repair remains unclear. Therefore, our study aims to determine the incidence of complications and associated risk factors among patients with non-proximal hypospadias after surgery.

## Materials and Methods

### Literature Search

Our methods were in accordance with the Preferred Reporting Items for Systematic Reviews and Meta-Analyses (PRISMA) guidelines (5). A systematic search of the PubMed, Embase, and Cochrane Library CENTRAL for the relevant published studies, which reported the postoperative outcomes of patients with non-proximal hypospadias, was conducted in June 2020. Year of publication was restricted to last three decades, from January 1990 to June 2020. The strategy used for searching was (Coronal hypospadias OR subcoronal hypospadias OR glanular hypospadias OR midshaft hypospadias OR penile hypospadias OR distal hypospadias OR anterior hypospadias) AND (Urethrocutaneous fistula OR urethral stricture OR meatal stenosis OR complication OR urethral diverticulum OR dehiscence OR meatal retraction). References from all the included studies and other relevant literature were also manually reviewed to identify additional eligible studies. This search was restricted to articles published in English. We contacted the authors to obtain extra information via e-mail, as necessary.

### Study Selection

A study was included in this systematic review when the following criteria were met: (1) case series, observational studies, or randomized controlled trials (RCTs), which were published on peer-reviewed journals and reported the postoperative outcomes of patients with distal and mid-penile hypospadias; (2) studies that reported postoperative outcomes of primary repair; and (3) studies that involved patients under 16 years of age.

A study was excluded in this systematic review when the following criteria were met: (1) multiple studies were based on the same data; (2) sample size of study was <100 cases; (3) studies involved patients with prior repair of hypospadias; (4) studies involved patients with isolated chordee without abnormal location of meatus; and (5) studies involved patients with proximal hypospadias. Reviews, letters, conference abstract, case reports, and animal experiments were also excluded. Only the study with most complete set of data was included when several studies were based on the same database and time period. Two reviewers (YWu and JW) searched and screened all the studies independently, and any disagreements on the eligibility of studies were resolved by consensus with a third reviewer (YWe).

### Data Extraction and Definition of Variables

Data were extracted by both reviewers (YWu and JW) independently, and any disagreement was resolved by consensus with the help of a third reviewer (JW). A standardized extraction form in an Excel spreadsheet was used. The following information was extracted: (1) baseline characteristics of included studies: first author, publication year, study area, types of study design, types of hypospadias, sample size, age, types of repair, chordee, and hormonal therapy prior to repair; (2) primary outcome: UCF; and (3) secondary outcomes: meatal stenosis, urethral stricture, meatal retraction, urethral diverticulum, wound infection and dehiscence, foreskin necrosis, urethral diverticulum, overall complications, and reoperation. Length of hospital stay, urethral stent indwelling, antibiotics usage, and penile dressing were also involved as the perioperative variables.

Types of hypospadias were recorded as distal, mid-penile, and proximal hypospadias according to the locations of meatus, and non-proximal hypospadias included distal and mid-penile types. Moreover, distal hypospadias included glanular, coronal, and subcoronal hypospadias. The mid-penile type was defined as meatus in the penile shaft. Proximal hypospadias included penoscrotal, scrotal, and perineal hypospadias. Hormonal therapy involved either local application or intramuscular injection of testosterone. Wound dehiscence included glanular or prepuce dehiscence. Meatal retraction was defined as regression of meatus without wound dehiscence. Overall complications were defined as all encountered complications following surgery. In patients who underwent day-case surgery, hospitalization was not needed. Meatal dilation due to stenosis without surgical repair was not considered as reoperation.

### Quality Assessment and Risk of Bias

Quality assessment and risk of bias were performed by two reviewers (LH and TZ) independently, and any divergences on the assessed quality were resolved with a third reviewer (SW) by consensus. The risk of bias of single-arm case series was evaluated using the method published by Murad et al. (6). The risk of bias of comparative non-RCTs was evaluated with the ROBINS-I tool (7). The risk of bias of RCTs was evaluated using the Cochrane Risk of Bias Tool (8).

### Statistical Analysis

Statistical analyses were conducted using Stata 12.0 (Stata Corp, Texas, United States). The χ^2^-Q statistics and the I^2^ statistics were used to assess the heterogeneity, with *I*^2^ > 50% indicating significant heterogeneity. If only the median value and range were available in our included studies, the formulas provided by Hozo et al. (9) would be used to estimate the mean value. A *p* < 0.05 was considered statistically significant for all analyses in our study.

For single-arm case series, we performed meta-analysis of prevalence to pool postoperative morbidity. We utilized a random-effects model for meta-analysis due to the likelihood of inter-observational heterogeneity. The Freeman–Tukey double arcsine transformation was used to adjust prevalence. Adjusted estimates were pooled by the inverse variance method of DerSimonian–Laird. Subgroup and meta-regression analyses were used to explore risk factors associated with primary outcome. Subgroup analysis was conducted in terms of study area, types of repair, types of hypospadias, hospitalization, mean age, length of antibiotics usage, penile dressing, urethral stent indwelling, and follow-up. Meta-regression analysis was performed with random models using aforementioned continuous variables.

For two-arm comparative studies (i.e., case-controlled studies, cohort, or RCT), we performed meta-analysis to evaluate outcomes of two compared interventions. Relative risk (RR) or odds ratio (OR) was employed in this setting. If the *I*^2^ statistic was over 50%, a random-effects model was adopted; otherwise, a fixed-effects model of analysis was conducted.

## Results

### Characteristics of Included Studies and Systematic Review

**Figure 1:**
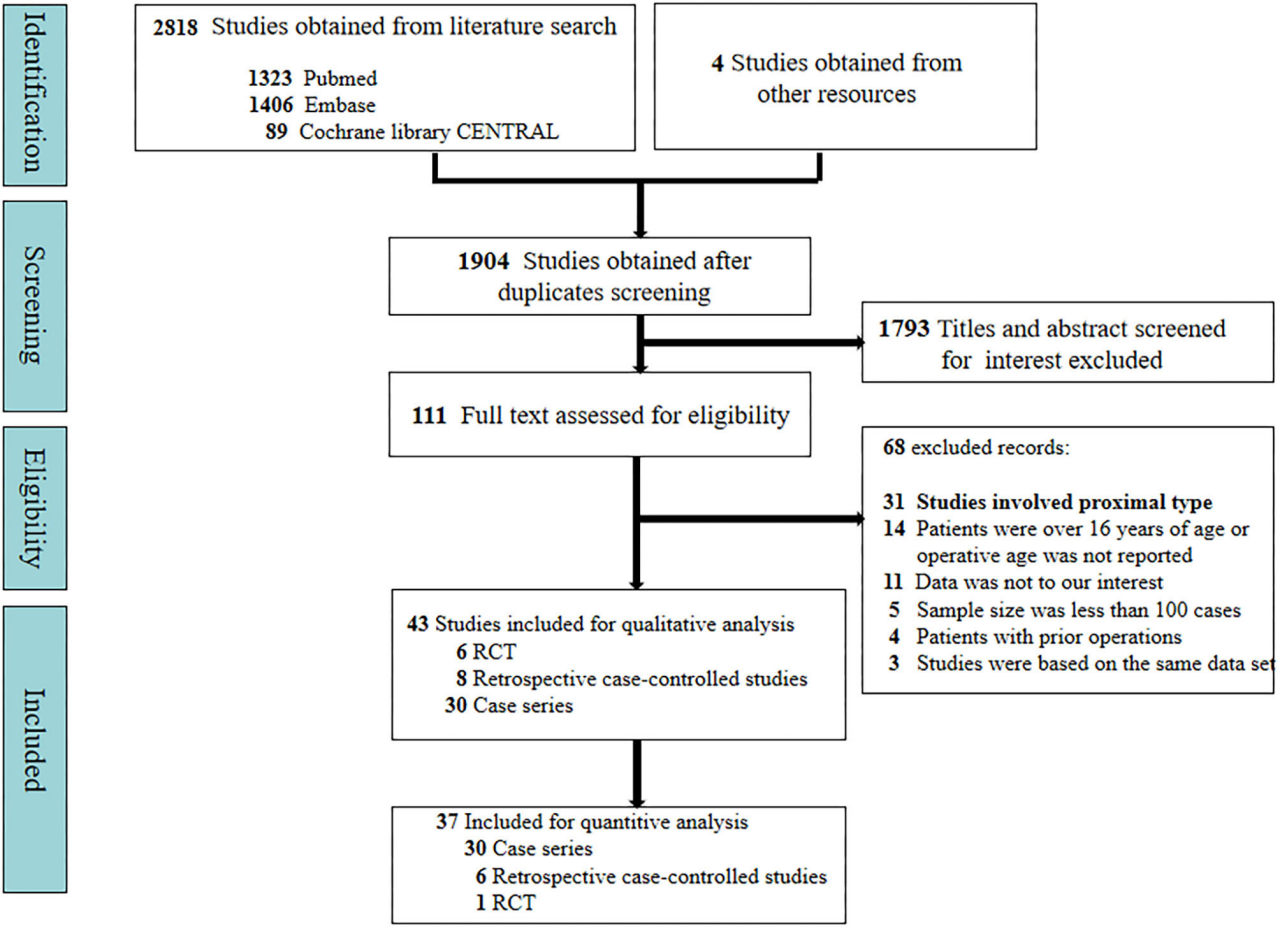
Flow diagram according to the preferred reporting items for systematic reviews and meta-analyses (PRISMA) protocol recommendations.

A total of 2,818 studies were obtained initially from the electronic databases, and four papers were further identified manually on reference lists of the retained studies. After duplicates and relevance in titles and abstracts were screened, only 111 studies were available for the full-text evaluation. Sixty-eight studies were excluded after full-text evaluation. Eventually, this review was based on 44 studies (10–53), which encompassed 30 case series, 8 case-controlled studies, and 6 RCTs. A total of 10,666 patients were involved in this study. All of our included studies were included for qualitative review. Quantitative synthesis was based on 30 case series, 6 case-controlled studies, and 1 RCT. A flowchart depicting the search strategy is shown in the Figure 1.

Meatal advancement and glanuloplasty (MAGPI), tubularized incised plate repair (Snodgrass technique), and meatal-based flip-flap repair (Mathieu technique) were mainly performed in distal and mid-penile hypospadias. Among our included case series, 26 studies reported the use of single technique, whereas 4 studies adopted divergent surgical approaches (Supplemental Table 1). Only two studies (11, 23) reported the use of hormonal therapy before surgery. UCF was the most common complication after surgery. In contrast, urethral stricture and wound infection were rarely reported. None of our included case series reported foreskin necrosis and urethral diverticulum.

Four case-controlled studies (40–43) compared postoperative outcomes in patients with or without urethral stent after surgery. Two case-controlled studies and one RCT compared foreskin preservation vs. circumcision in distal hypospadias repair (45–47). Five RCTs and one case-controlled study (48–53) compared variant modifications of Snodgrass repair.

### Primary Outcome

#### Urethrocutaneous Fistula

**Figure 2:**
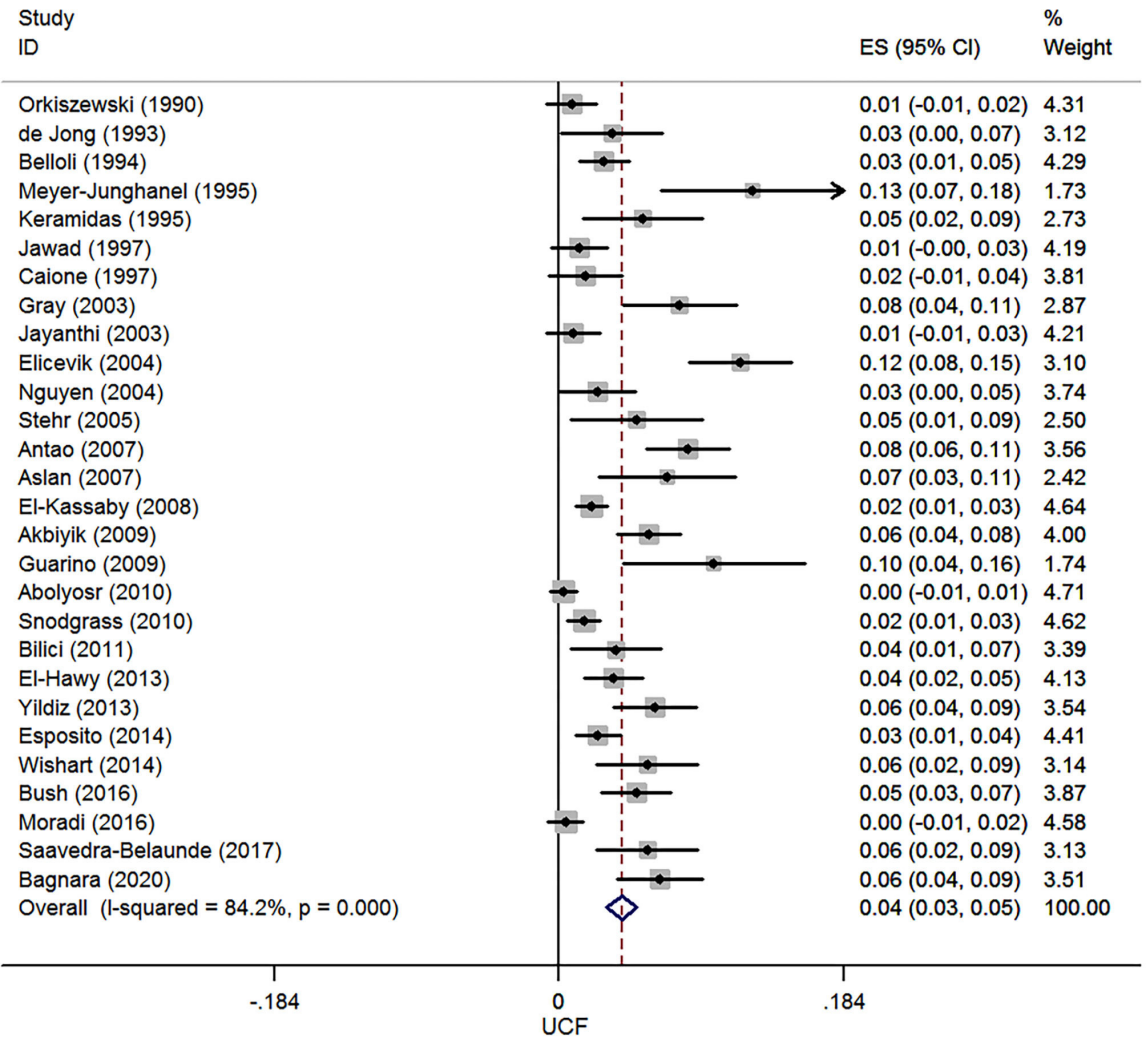
Forest plot of incidence of urethrocutaneous fistula following hypospadias repair.

A total of 28 case series reported UCF after hypospadias repair, and 7,485 patients were involved in this meta-analysis. The pooled estimate of incidence of UCF was 4.0% (95% CI, 3.1–5.0%, *I*^2^ = 84.2%, Figure 2).

### Secondary Outcomes

#### Meatal Stenosis

**Figure 3:**
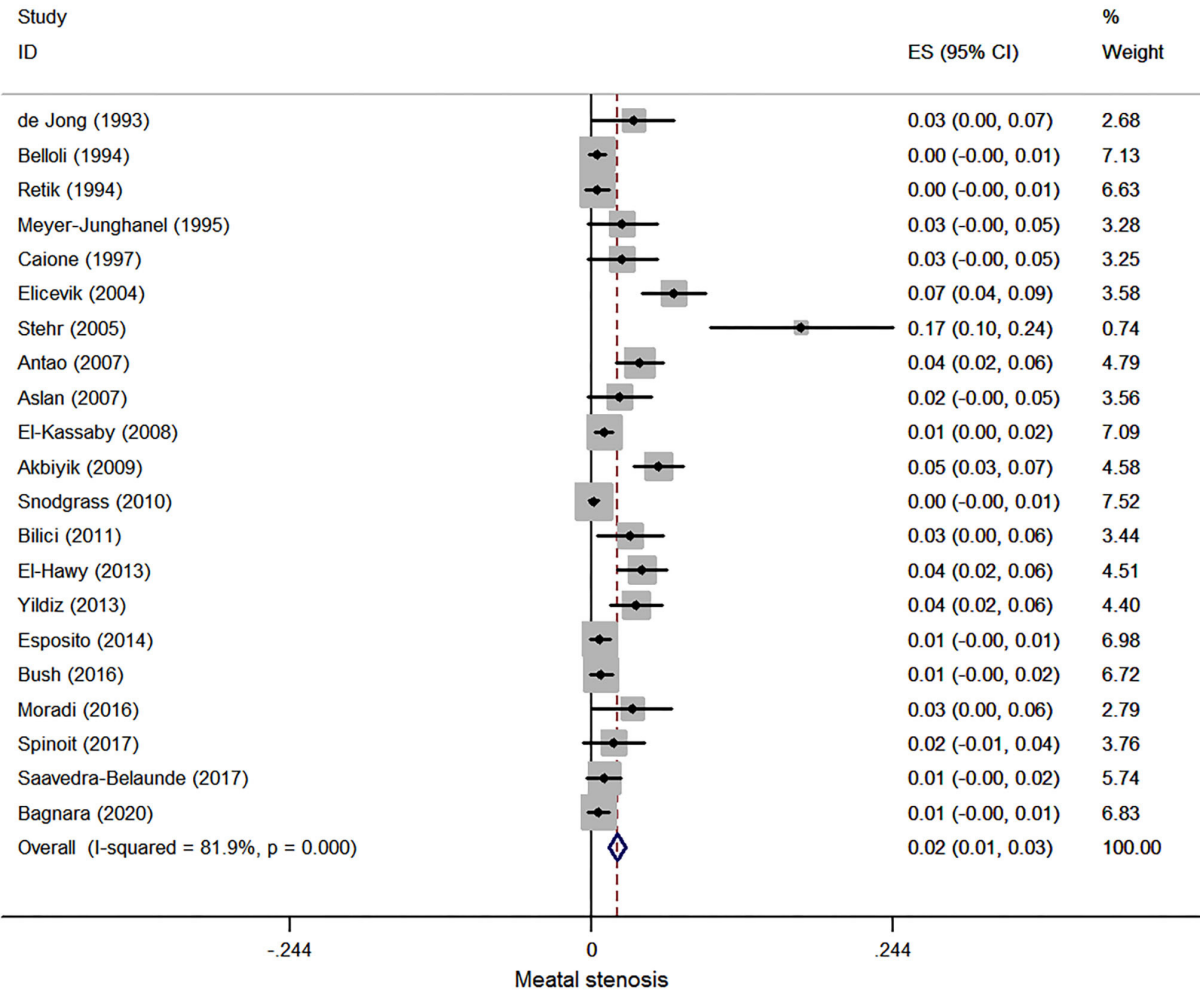
Forest plot of incidence of meatal stenosis following hypospadias repair.

Twenty-one case series reported meatal stenosis after hypospadias repair, and 6,306 patients were involved in this meta-analysis. The pooled estimate of incidence of meatal stenosis was 2.1% (95% CI, 1.4–2.8%, *I*^2^ = 81.9%, Figure 3).

#### Urethral Stricture

In patients with distal and mid-penile hypospadias, postoperative urethral stricture was scarce, and only two case series (20, 32) reported this complication with an incidence of 0.8 and 0.7%.

#### Meatal Retraction

**Figure 4:**
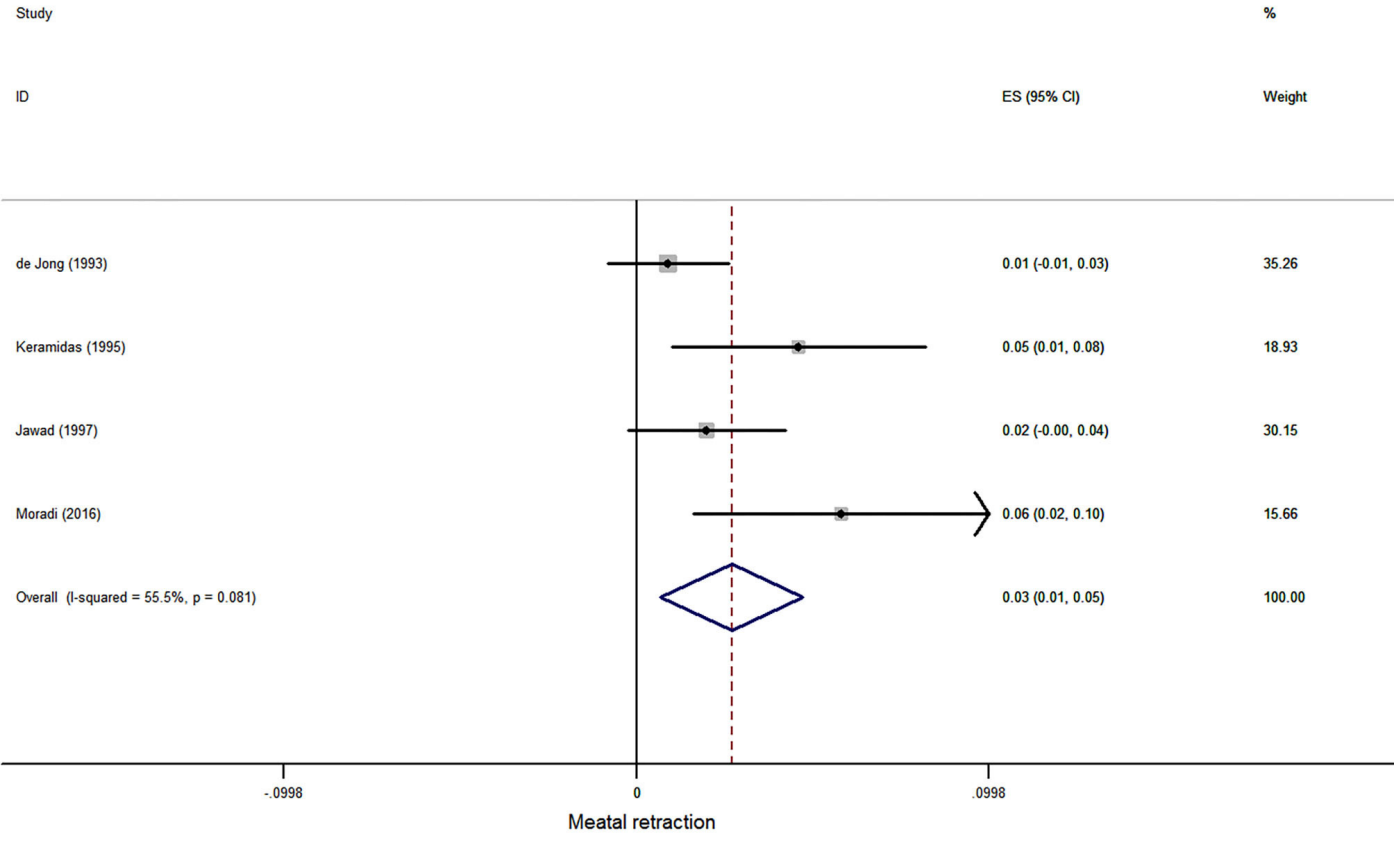
Forest plot of incidence of meatal retraction following hypospadias repair.

Three case series reported meatal retraction after hypospadias repair, and 380 patients were involved in this meta-analysis. The pooled estimate was 3.4% (95% CI, 0.1–6.6%, *I*^2^ = 70.3%, Figure 4).

#### Wound Dehiscence

**Figure 5:**
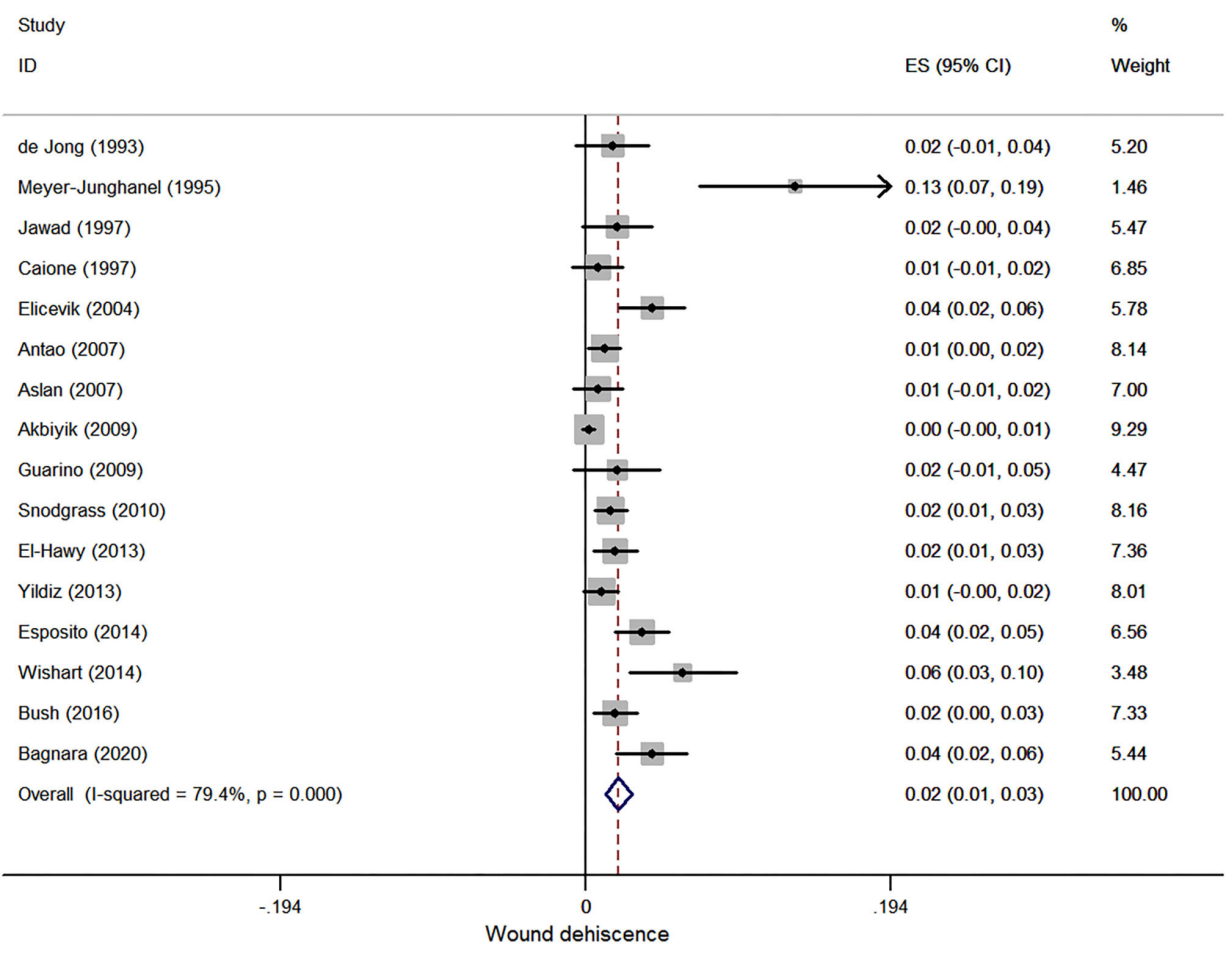
Forest plot of incidence of wound dehiscence following hypospadias repair.

Sixteen case series reported wound dehiscence after hypospadias repair, and 4,648 patients were involved in this meta-analysis. The pooled estimate was 2.1% (95% CI, 1.3–2.9%, *I*^2^ = 79.4%, Figure 5).

#### Overall Complications

**Figure 6:**
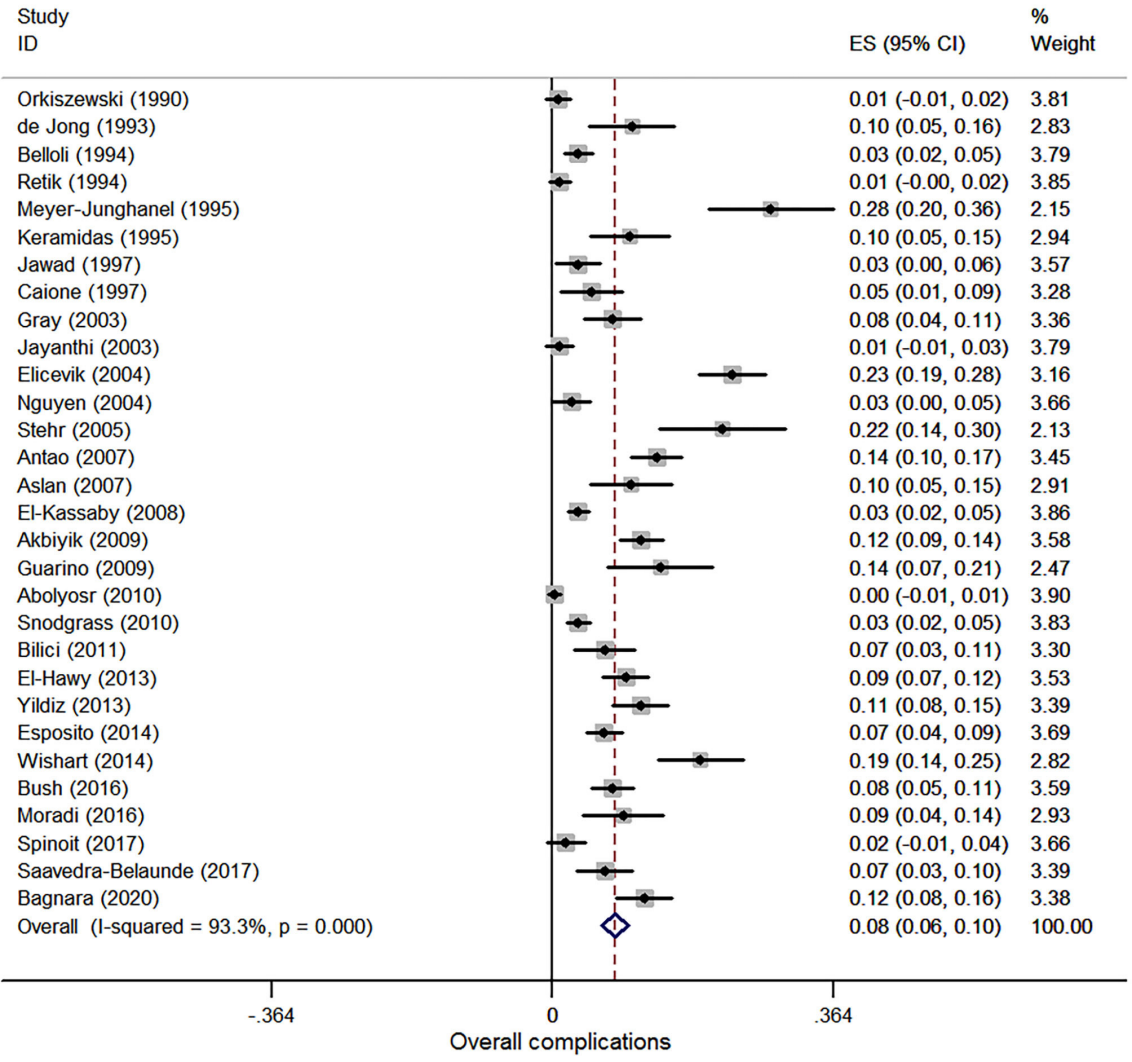
Forest plot of incidence of overall complications following hypospadias repair.

Thirty studies were involved in the meta-analysis of overall complications, and a total of 7,474 patients were included. The pooled estimate of overall complications was 8.0% (95% CI, 6.3–9.8%, *I*^2^ = 93.3%, Figure 6).

#### Re-operation

**Figure 7:**
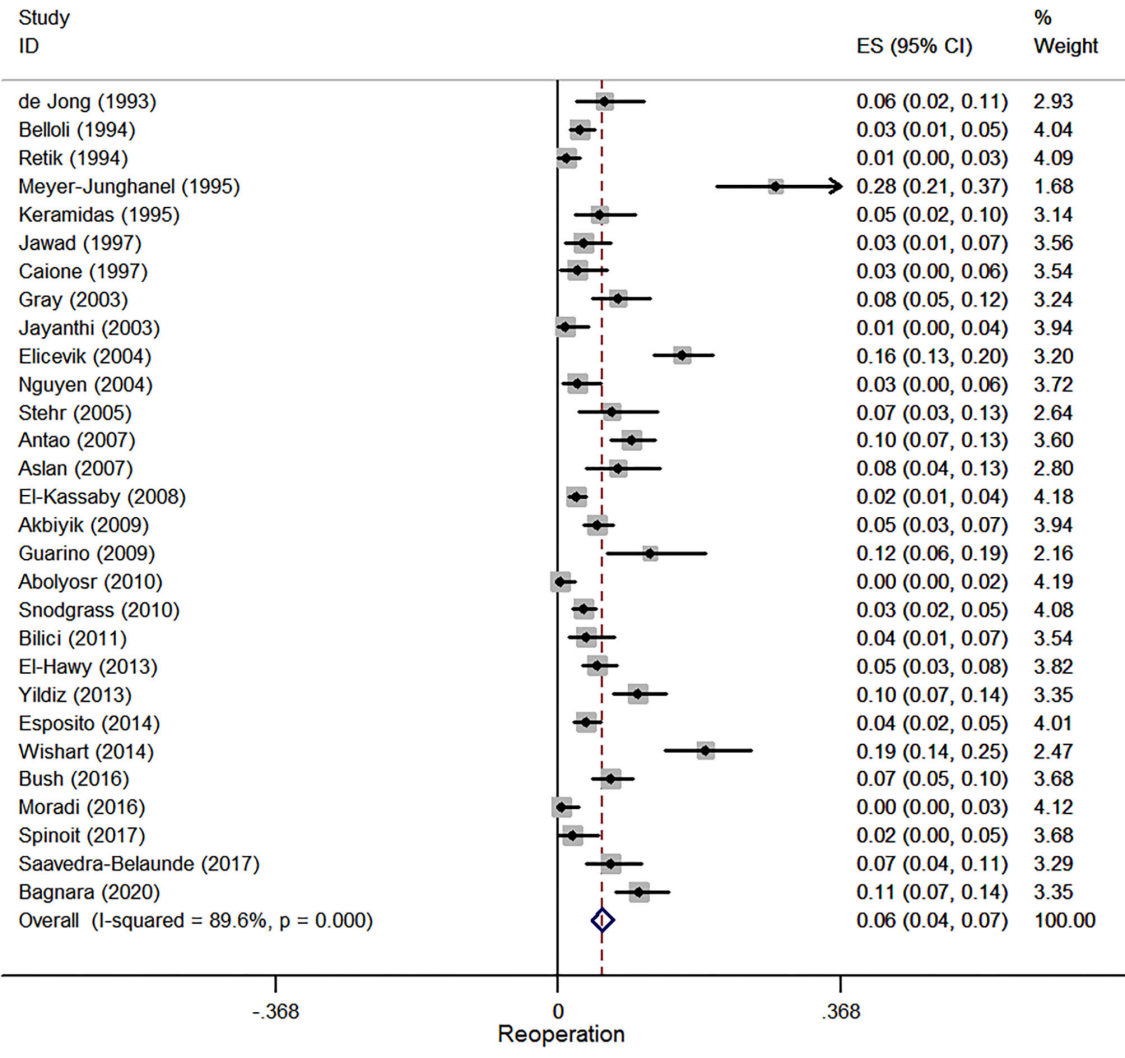
Forest plot of incidence of reoperation following hypospadias repair.

Twenty-nine studies were involved in the meta-analysis of reoperation, and a total of 7,356 patients were included. The pooled estimate of reoperation was 5.7% (95% CI, 4.4–7.1%, *I*^2^ = 89.6%, Figure 7).

### Sub-group Analysis

Subgroup analysis (Supplemental Table 2) was applied to our primary outcome in terms of study area, types of repair, types of hypospadias, hospitalization, operative age, follow-up, length of antibiotics usage, penile dressing, and urethral stent indwelling. Day-case surgery, older operative age, and longer length of antibiotics usage, stent indwelling, penile dressing, and follow-up were associated with a higher incidence of UCF. Additionally, as the percentage of mid-penile hypospadias increased, so did the incidence of UCF. Snodgrass repair, caudal block, and Vicryl suture were associated with a lower incidence of UCF. Our involved studies mainly originated from three continents, and incidence of UCF varied due to regional differences.

### Meta Regression Analysis

**Figure 8:**
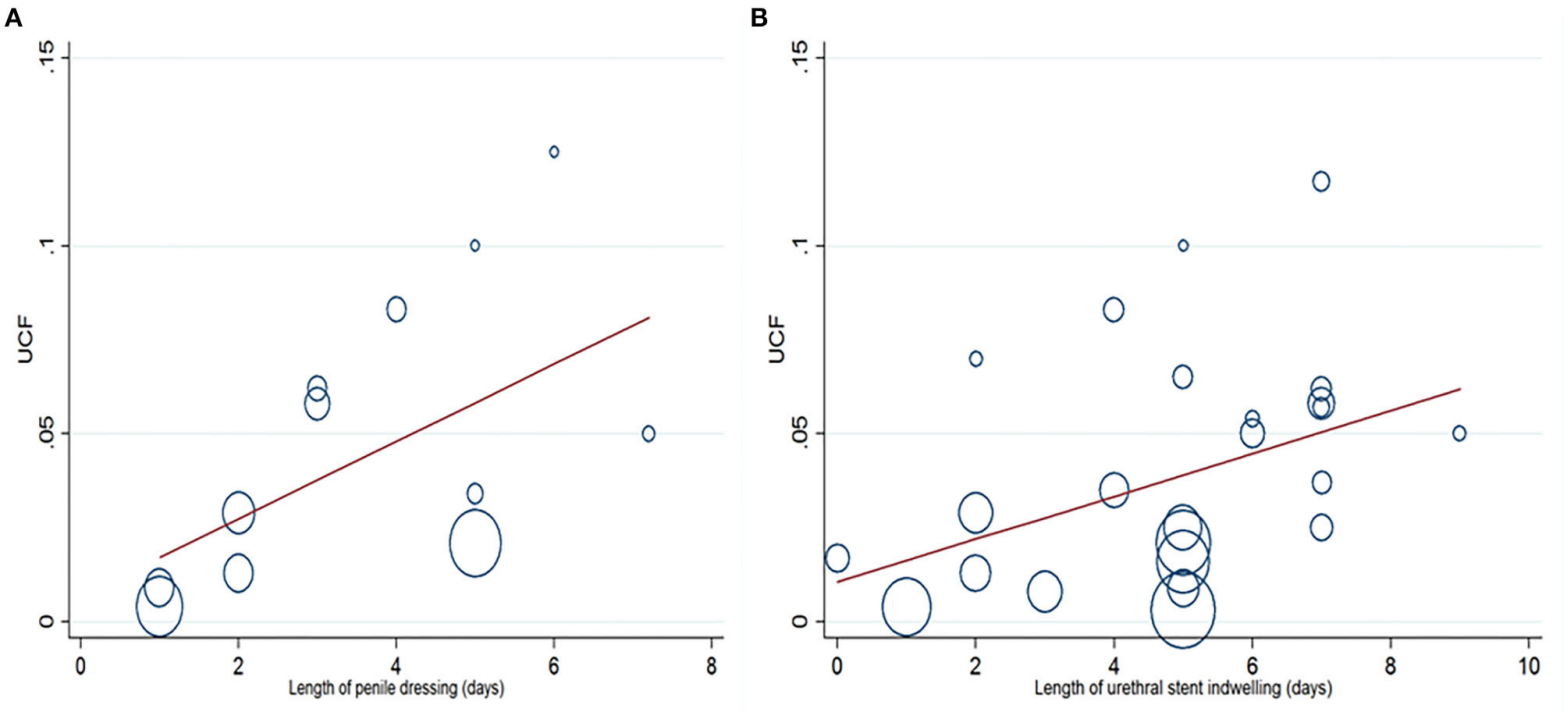
Univariate meta-regression analysis indicates that length of penile dressing **(A)** and urethral stent indwelling **(B)** are the risk factors leading to urethrocutaneous fistula (UCF).

Meta-regression analysis (Supplemental Table 3) was also applied to UCF in terms of follow-up, age, length of antibiotics usage, penile dressing, associated chordee, and urethral stent indwelling. In the univariate meta-regression analysis, length of penile dressing (p = 0.048) and urethral stent indwelling (*p* = 0.036) were the risk factors leading to UCF (Figure 8). However, a further multivariate meta-regression analysis did not identify any independent risk factors for UCF.

### Stent vs. Stentless in Non-proximal Hypospadias

Four case-controlled studies (40–43) compared outcomes in patients with or without urethral stent after surgery, and a total of 802 patients were involved. Snodgrass and Mathieu technique were involved for distal or mid-penile hypospadias repair. Meta-analysis results indicated that no differences were found between stent and stentless group regarding incidences of UCF (OR, 0.589; 95% CI, 0.267–1.297; *p* = 0.189; *I*^2^ = 27.9%), meatal stenosis (OR, 0.880; 95% CI, 0.318–2.437; *p* = 0.805; *I*^2^ = 0%), and overall complications (OR, 0.695; 95% CI, 0.403–1.199; *p* = 0.191; *I*^2^ = 46.3%, Supplemental Figure 1).

### Foreskin Preservation vs. Circumcision in Non-proximal Hypospadias

Two case-controlled studies (46, 47) and one RCT (45) compared foreskin preservation with circumcision in distal hypospadias repair, and 650 patients were included. Meta-analysis results indicated that no differences were found between foreskin preservation and circumcision groups regarding incidences of UCF (OR, 0.558; 95% CI, 0.176–1.769; *p* = 0.322; *I*^2^ = 78.1%) and wound dehiscence (OR, 0.743; 95% CI, 0.164–3.357; *p* = 0.699; *I*^2^ = 57.2%). Meta-analysis of overall complication was not performed due to significant heterogeneity *(I*^2^ = 81.8%). The RCT performed by ElGanainy et al. (45) found that the overall complications between foreskin preservation and circumcision groups were similar (*p* = 0.967).

### Snodgrass Technique and Its Variants in Non-proximal Hypospadias

Five RCTs and one case-controlled study compared variant modifications of Snodgrass repair (48–53). Three RCT (49, 52, 53) compared dartos flap with non-dartos flap repair. No difference was found regarding UCF (RR, 0.637; 95% CI, 0.376–1.079; *p* = 0.094; *I*^2^ = 46.6%, Supplemental Figure 2) and wound dehiscence (RR, 2.032; 95% CI, 0.888–4.650; *p* = 0.093; *I*^2^ = 0%, Supplemental Figure 3). One case-controlled study (51) found that Snodgrass repair combined with a meatus-based ventral dartos flap or dorsal dartos flap was similarly effective with respect to UCF. Other two RCTs (48, 50) showed a lower morbidity with the use of platelet-rich plasma layer (48) and lateral augmentation (50) in non-proximal hypospadias surgery.

### Risk of Bias

Assessment of risk of bias among case series was conducted in four domains with eight explanatory questions (Supplemental Table 4). ROBINS-I tool was used to evaluate comparative non-RCT studies, and all of them were graded as moderate risk of bias (Supplemental Table 5). Six domains of RCT were assessed using the Cochrane Risk of Bias Tool, and six RCTs received a high risk of bias rating (Supplemental Table 6).

## Discussion

In this study, we found that the most common complication following hypospadias repair was UCF with an incidence of 4.0%. We also investigated other common complications such as meatal retraction and meatal stenosis with an incidence of 2.7 and 2.1%, respectively. Complications such as urethral stricture, foreskin necrosis, wound infection, and urethral diverticulum were rarely reported. Overall complications after hypospadias repair occurred in 8.0% of our population.

To explore the risk factors associated with occurrence of UCF, subgroup analysis was performed. We found that day-case surgery, increased operative age, mid-penile hypospadias, prolonged length of antibiotics usage, stent indwelling, and penile dressing might contribute to UCF occurrence after non-proximal hypospadias repair. Snodgrass repair, caudal block, and Vicryl suture might decrease incidence of UCF. Increased follow-up duration was also associated with a higher incidence of UCF, which warranted a sufficient period of follow-up after non-proximal hypospadias repair. Through a univariate meta-regression analysis, we observed that prolonged length of urethral stent indwelling and penile dressing were risk factors for UCF. However, a further multivariate meta-regression analysis did not identify any independent risk factors for UCF. This might suggest additional attentions from surgeons when length of stent indwelling and penile dressing were prolonged.

Meta-analysis of four case-controlled studies revealed no differences between stent and stentless group in terms of UCF, meatal stenosis, and overall complications. The absence of urethral stent did not compromise the postoperative recovery in distal and mid-penile hypospadias repair. This was similar to the findings of a recent systematic review (54). Furthermore, a decreased incidence of bladder spasm and stent-related urinary infection using stentless repair was also reported (40, 55). However, based on our included studies, we were not able to confirm a definite correlation between stent indwelling and bladder spasm or urinary infection. More prospective studies with sufficient participants should be conducted in this regard. We also compared foreskin preservation with circumcision in distal and mid-penile hypospadias. Similarly, we did not identify significant differences regarding UCF, wound dehiscence, and overall complications. This was also consistent with conclusions of several prior studies (56, 57), although UCF seemed to be less visible with foreskin preservation.

In our study, only one case-controlled study (44) compared Mathieu with Snodgrass technique. They found that Mathieu technique entailed a higher incidence of UCF. However, in recent systematic reviews (58, 59), no significant differences were found in terms of UCF. Therefore, further RCTs should be designed to explore the clinical outcomes between these two procedures.

The European Association of Urology (EAU) guideline recommended optimal age for hypospadias repair between 6 and 18 months of age (60). Primary hypospadias repair at a later stage could be associated with more complications such as infection, hematoma, and wound dehiscence due to urethral secretion and nocturnal erection. In our study, we regarded 12 months of age as the cutoff time in the subgroup analysis. A positive correlation between age and incidence of UCF was also noticed (2.2 vs. 4.6%), although meta-regression analysis did not reach statistical difference.

## Limitations

Limitations in our study are acknowledged here. First, our study was mainly based on retrospective studies, and hence, our meta-analysis was of low quality. Second, long-term assessment of functional, cosmetic, and sexual outcomes following hypospadias repair was absent based on our included studies. Besides, there have been no available standardized questionnaires for the assessment of psychosexual function after hypospadias repair. Third, we could not identify whether other perioperative factors such as center volume, surgeons' experiences, and preoperative hormonal therapy had significant impact on our primary outcomes due to limited data. Fourth, a few self-designed technique and variants with a limited number of participants did not meet our inclusive criteria; therefore, our results might not be appropriate for all non-proximal conditions. Fifth, although Murad and ROBINS-I tools were the most accepted approaches for quality evaluation of non-RCT studies, major drawbacks of hypospadias literature such as limited follow-up periods, no disclosure of lost to follow-up, single-surgeon outcomes vs. team outcomes, and a core outcome set of hypospadias (61) were not well-assessed. Last, since this study was a meta-analysis of proportion, the heterogeneity was significant. At the same time, due to the lack of comparative groups in our meta-analysis of proportion, our results were less conclusive.

## Conclusions

In conclusion, the most common complication following non-proximal hypospadias repair is UCF with an incidence of 4.0%. Urethral stricture and diverticulum, foreskin necrosis, and wound infection after surgery are rare. Incidence of overall complications is 8.0%. Distal hypospadias repair without stent indwelling is not likely to compromise the postoperative outcomes. However, due to the lack of comparative group and high heterogeneity in the quantitative analyses, our results may not be appropriate for all non-proximal conditions. More RCTs should be designed to explore the differences between different approaches and the potential risk factors for complications following hypospadias repair.

## Data Availability Statement

The raw data supporting the conclusions of this article will be made available by the authors, without undue reservation.

## Author Contributions

YWu and JW carried out project development, collected the data, and wrote the manuscript. TZ, YWe, and LH collected the data. YWu, TL, and XL wrote the manuscript. GW and SW performed critical revision. All authors contributed to the article and approved the submitted version.

## Conflict of Interest

The authors declare that the research was conducted in the absence of any commercial or financial relationships that could be construed as a potential conflict of interest.

## Supplementary Material

Supplemental Figure 1 Forest plot of overall complications in stent vs. stentless repair.

Supplemental Figure 2 Forest plot of urethrocutaneous fistula (UCF) in dartos flap vs. non-dartos flap repair.

Supplemental Figure 3 Forest plot of wound dehiscence in dartos flap vs. non-dartos flap repair.

Supplemental Table 1 Characteristics of included studies.

Supplemental Table 2 Subgroup analysis of primary outcome among case series.

Supplemental Table 3 Meta-regression analysis of primary outcome based on case series and pre-specified variables.

Supplemental Table 4 Assessment of risks of bias of case series.

Supplemental Table 5 Assessment of risks of bias of comparative non-RCT studies.

Supplemental Table 6 Assessment of risks of bias of RCT.
