# Supplementary figures and images for: Complications Following Primary Repair of Non-proximal Hypospadias in Children: A Systematic Review and Meta-Analysis
Source: Front Pediatr. 2020 Dec 9;8:579364. doi: 10.3389/fped.2020.579364 (PMC7756017; doi:10.3389/fped.2020.579364)

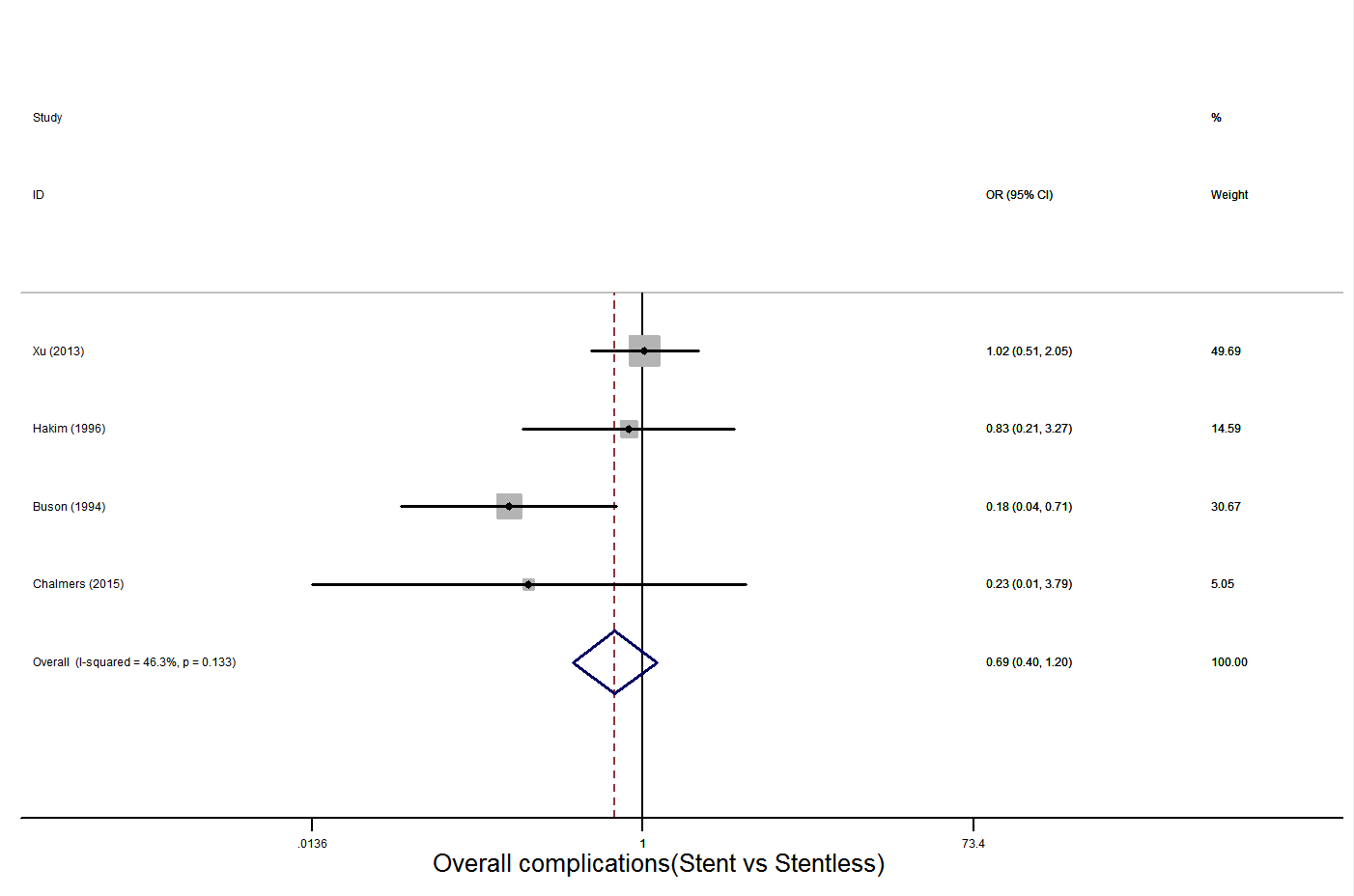

Supplement: Supplemental Figure 1 — Forest plot of overall complications in stent vs. stentless repair. [file Image_1.TIF]

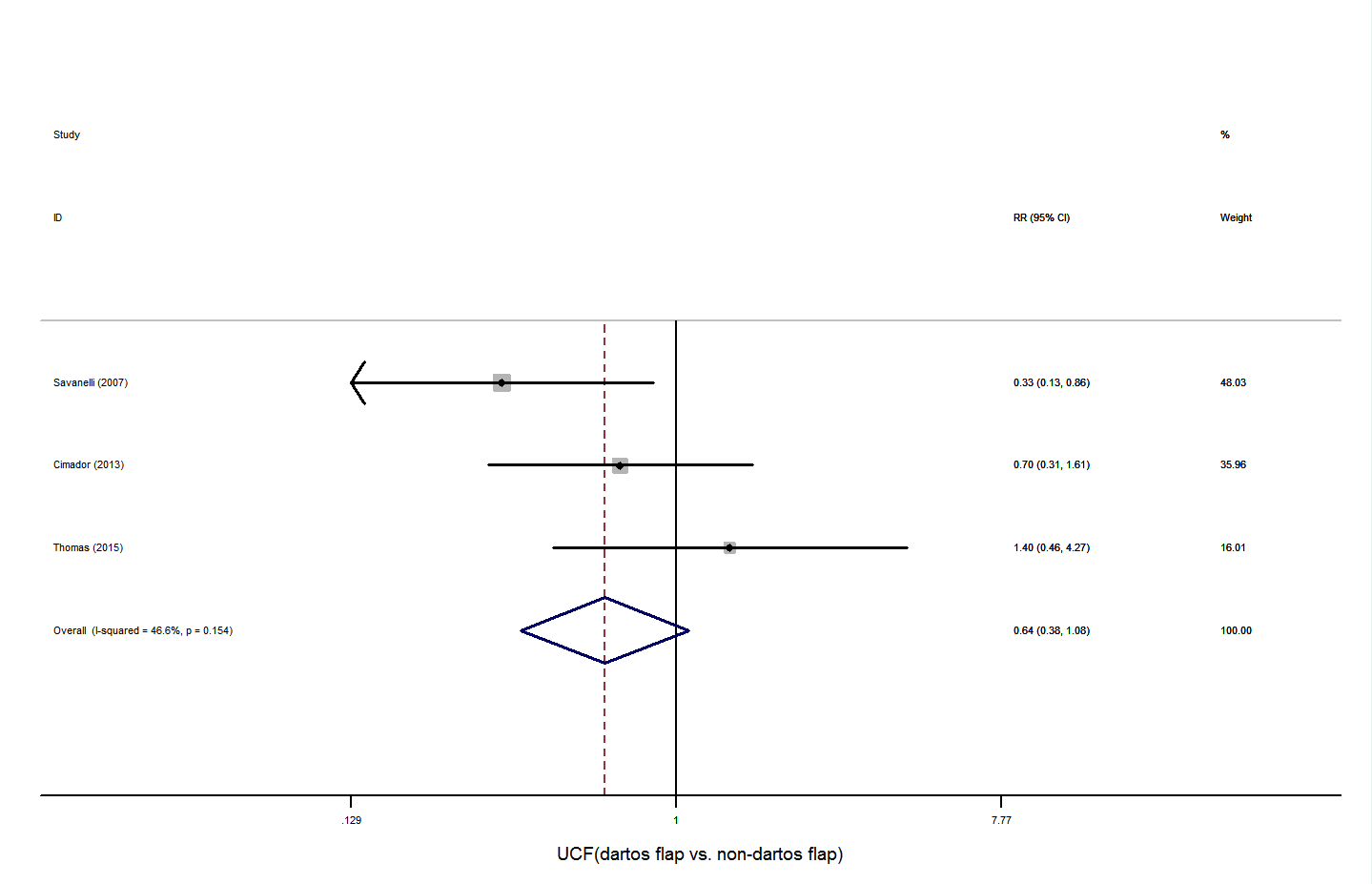

Supplement: Supplemental Figure 2 — Forest plot of urethrocutaneous fistula (UCF) in dartos flap vs. non-dartos flap repair. [file Image_2.TIF]

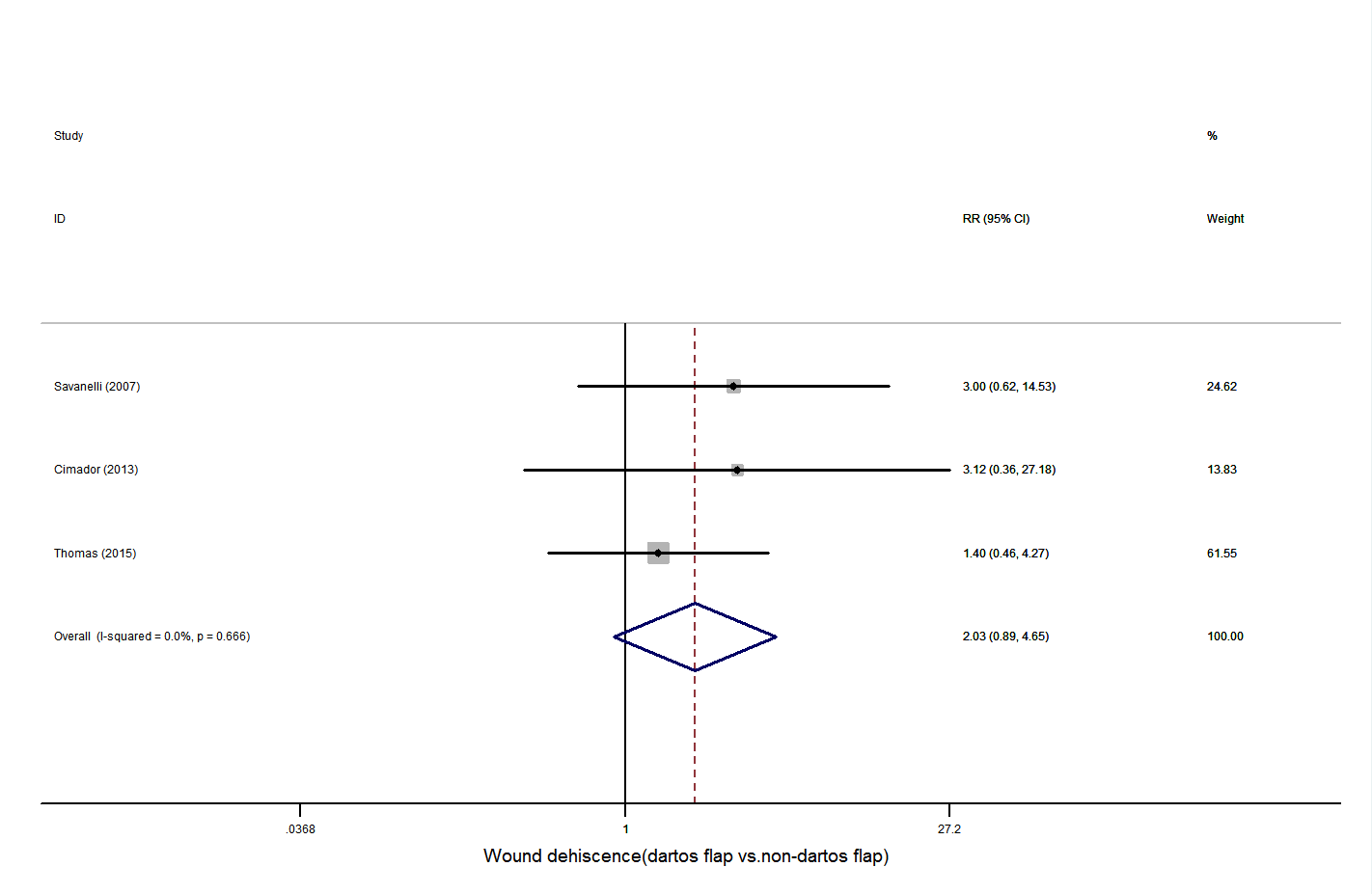

Supplement: Supplemental Figure 3 — Forest plot of wound dehiscence in dartos flap vs. non-dartos flap repair. [file Image_3.TIF]
