# Supplementary material for: Complications Following Primary Repair of Non-proximal Hypospadias in Children: A Systematic Review and Meta-Analysis
Source: Front Pediatr. 2020 Dec 9;8:579364. doi: 10.3389/fped.2020.579364 (PMC7756017; doi:10.3389/fped.2020.579364)
Supplement: Supplemental Table 1 — Characteristics of included studies. [file Table_1.DOC]

**Supplemental table 1 Characteristics of included studies**

| **Author, year and area** | **Patients(n)** | **Age*** | **Types of hypospadias (n)** | **Surgical technique** | **Follow-up*** | **Study design** |
| --- | --- | --- | --- | --- | --- | --- |
| Orkiszewski,1990, Poland | 118 | 12m-16y | Distal 118 | MAGPI/Mathieu/Duckett | NA | Case series |
| de Jong,1992, Netherlands | 116 | 1.4y | Distal | Mathieu | 2.5y | Case series |
| Belloli,1994,Italy | 408 | <6y | Mid-penile | Mathieu | 3m | Case series |
| Retik,1994,USA | 204 | 5m-16y | Distal | Mathieu | 6m-6y | Case series |
| Meyer-Junghanel, 1995, Germany | 120 | 3.7y | Distal 114, mid-penile 6 | Mathieu | 9m | Case series |
| Keramidas,1995,Greece | 130 | 5.3y | Distal | UAGP | NA | Case series |
| Caione, 1997, Italy | 118 | 2.5y | Distal | MAGPI | NA | Case series |
| Jawad,1997,Saudi Arabia | 153 | 1.7y | Distal | MAGPI/Urethral mobilization and advancement | 3-12m | Case series |
| Gray,2003,UK | 205 | 3.3y | Distal | GAP | >10y | Case series |
| Jayanthi,2003,USA | 110 | 0.8y | Distal | Snodgrass | 9.4m | Case series |
| Elicevik,2004, Turkey | 360 | 4.3y | Mid-penile | Snodgrass | 6m-5y | Case series |
| Nguyen,2004,USA | 159 | 20m | Distal | Snodgrass | 8m | Case series |
| Stehr,2005, Germany | 100 | 3.5y | Distal | Snodgrass | 2y | Case series |
| Antao,2007,UK | 408 | 1.1y | Distal | MAGPI/Snodgrass/Mathieu/Barcat | 11m | Case series |
| Aslan,2007,Turkey | 128 | 3.4y | NA | Snodgrass | 1.9y | Case series |
| El-Kassaby,2008,Egypt | 764 | 2y | Distal | Snodgrass | 1.5y | Case series |
| Akbiyik,2009,Turkey | 496 | 3.6y | Distal 389, mid-penile 107 | Snodgrass | 2y | Case series |
| Guarino,2009, Italy | 100 | 1y | Distal | Snodgrass | 2y | Case series |
| Abolyosr,2010, Egypt | 156 | 4.5y | Distal 98, mid-penile 58 | Snodgrass | 2y | Case series |
| Snodgrass,2010, USA | 551 | 0.3y | Distal | Snodgrass | 8.2m | Case series |
| Bilici,2011, Turkey | 161 | 2.4y | Distal 96, mid-penile 63 | Snodgrass | 1y | Case series |
| El-Hawy,2013,Egypt | 369 | 7y | Distal | Snodgrass | 5y | Case series |
| Yildiz,2013,Turkey | 307 | 5.6y | Distal 269, mid-penile 38 | Snodgrass | 8m | Case series |
| Esposito,2014,Italy | 445 | 1.8y | Distal | MAGPI/Snodgrass | NA | Case series |
| Wishart,2014, Canada | 193 | 1.5y | Distal | Snodgrass | NA | Case series |
| Bush,2016,USA | 361 | 1.5y | NA | Snodgrass | 3-9m | Case series |
| Moradi,2016,Iran | 120 | 3.2y | Distal | MAGPI | 2y | Case series |
| Spinoit,2017,UK | 112 | 2y | Distal | Snodgrass | 1.8y | Case series |
| Saavedra-Belaunde,2017,Puerto Rico | 192 | 11m | Distal | Snodgrass | 4y | Case series |
| Bagnara,2020,Italy | 310 | 2y | Distal | Snodgrass/Duplay | 8.1y | Case series |
| Xu,2013,China | 103  151 | 4.2y  4.5y | Distal 65, mid-penile 38  Distal 102, mid-penile 49 | Snodgrass+stent  Snodgrass+stentless | 1.8y  2y | Case-controlled study |
| Chalmers,2015,USA | 89  21 | 0.6y  0.6y | Distal | Snodgrass/MAGPI+stent  Snodgrass/MAGPI+stentless | 3m | Case-controlled study |
| Buson,1993, USA | 65  37 | 5m-13y  6m-9y | Distal | Mathieu+stent  Mathieu+stentless | NA | Case-controlled study |
| Hakim,1994,USA | 114  222 | 1.7y | Distal | Mathieu+stent  Mathieu+stentless | NA | Case-controlled study |
| ElGanainy,2012,Egypt | 86  67 | 2.9y  2.9y | Distal | Mathieu+Foreskin preservation  Mathieu+circumcision | NA | RCT |
| Rampersad,2017, Australia | 95  118 | 1.6y  1.2y | Distal | Mathieu+Foreskin preservation  Mathieu+circumcision | 2.5y  2.8y | Case-controlled study |
| Samuel,2002,UK | 211  170 | 1.5y  1.6y | Distal 365, mid-penile 16 | Mathieu  Snodgrass | 3.1y | Case-controlled study |
| Mahmoud,2019,Egypt | 90  90 | 2.3y | Distal 66, mid-penile 24  Distal 68, mid-penile 22 | Snodgrass+platelet plasma layer  Snodgrass+dartos flap | 2.3y | RCT |
| Cimador,2013, Italy | 57  73 | 1.8y | Distal | Snodgrass+dorsal preputial flap  Snodgrass+ventral dartos flap | 1.7y  1.5y | RCT |
| Jia,2016,China | 356 | 2.3y | Distal 327, mid-penile 129 | Snodgrass+dorsal preputial flap  Snodgrass+ventral dartos flap | NA | Case-controlled study |
| Thomas,2015,Turkey | 107  107 | 3.6y | Distal 181, mid-penile 33 | Snodgrass+dorsal preputial flap  Snodgrass | 2y | RCT |
| Elbakry,2016, Egypt | 349 | 2.6y | Distal | Snodgrass+lateral augmentation  Snodgrass | 1y | RCT |
| Manuele,2019,UK | 89  21 | 7m | Distal | Mathieu+Foreskin preservation  Mathieu+circumcision | 17m | Case-controlled study |
| Savanelli,2007,Italy | 65  65 | 26.9 m | Distal | Snodgrass+ventral dartos flap  Snodgrass+non-cover | 24m | RCT |

*MAGPI* Meatal advancement and glanduloplasty, *GAP* Glandular reconstruction and preputioplasty

* Data was presented with mean/median values or range
