# Supplementary material for: Complications Following Primary Repair of Non-proximal Hypospadias in Children: A Systematic Review and Meta-Analysis
Source: Front Pediatr. 2020 Dec 9;8:579364. doi: 10.3389/fped.2020.579364 (PMC7756017; doi:10.3389/fped.2020.579364)
Supplement: Supplemental Table 2 — Subgroup analysis of primary outcome among case series. [file Table_2.DOC]

**Supplemental Table 2 Subgroup analysis of primary outcome among case series**

| **Subgroups** | **Studies (n)** | **Pooled estimates of UCF (95% CI)** | **Heterogeneity (I^2^)** |
| --- | --- | --- | --- |
| **Study area** | | | |
| North America | 6 | 3.2%(1.6% to 4.8%) | 72.1% |
| Europe | 12 | 4.9%(3.2% to 6.6%) | 79.1% |
| Asia | 7 | 4.9%(2.1% to 7.7%) | 90.9% |
| **Operative age** | | | |
| ≤1 years | 3 | 2.2%(0.3% to 4.1%) | 69.4% |
| >1 years | 23 | 4.6%(3.4% to 5.8%) | 89.0% |
| **Type of hypospadias** | | | |
| Exclusively distal | 20 | 3.6%(2.5% to 4.6%) | 78.7% |
| Mid-penile involved | 6 | 4.9%(1.9% to 7.9%) | 93.1% |
| **length of urethral stent indwelling** | | | |
| <1 week | 17 | 2.8%(1.9% to 3.8%) | 81.1% |
| ≥1 week | 7 | 5.7%(3.7% to 7.8%) | 71.6% |
| **Hospitalization** | | | |
| Day-case | 3 | 6.5%(2.5% to 10.4%) | 73.0% |
| >1 day | 4 | 4.2%(1.5% to 6.9%) | 74.9% |
| **Length of antibiotics usage** | | | |
| <1 week | 6 | 3.7%(1.5% to 5.8%) | 90.0% |
| ≥1 week | 4 | 4.8%(2.2% to 7.3%) | 82.9% |
| **Length of penile dressing** | | | |
| <1 week | 11 | 4.0% (2.4% to 5.7%) | 86.0% |
| ≥1 week | 1 | 5.0%(0.7% to 9.3%) | - |
| **Surgical technique** | | | |
| Snodgrass(TIP repair) | 16 | 4.3%(3.0% to 5.6%) | 85.5% |
| Mathieu | 3 | 5.3%(1.2% to 9.5%) | 78.7% |
| Hybrid* | 5 | 3.7%(1.2% to 6.1%) | 87.5% |
| **Local anesthesia** | | | |
| Caudal block | 3 | 5.5% (4.1% to 7.0%) | 6.9% |
| Penile block | 3 | 5.8% (3.6% to 8.1%) | 0 |
| **Vicryl/PDS/PGA suture** | | | |
| Vicryl | 7 | 1.5% (1.0% to 1.9%) | 89.4% |
| PDS | 6 | 3.7% (2.8% to 4.7%) | 76.8% |
| PGA | 4 | 4.8% (3.2% to 6.5%) | 49.0% |
| **Follow-up** | | | |
| <6 months | 4 | 3.7%(1.7% to 5.7%) | 74.2% |
| 6-12 months | 6 | 4.1%(1.7% to 6.6%) | 86.0% |
| >12 months | 13 | 4.8%(3.1% t0 6.5%) | 89.1% |

*UCF* Urethrocutaneous fistula; *TIP* Tubularized incised urethral plate urethroplasty

*Vicryl* Polyglactin suture; *PDS* Polydioxanone suture; *PGA* Polyglycolic acid suture

*Hybrid subgroup involved studies that reported divergent surgical technique
