# Supplementary material for: Complications Following Primary Repair of Non-proximal Hypospadias in Children: A Systematic Review and Meta-Analysis
Source: Front Pediatr. 2020 Dec 9;8:579364. doi: 10.3389/fped.2020.579364 (PMC7756017; doi:10.3389/fped.2020.579364)
Supplement: Supplemental Table 3 — Meta-regression analysis of primary outcome based on case series and pre-specified variables. [file Table_3.DOC]

**Supplemental table 3** Meta-regression analysis of primary outcome based on case series and pre-specified variables

| **Univariate** | | | | **Multivariate** | | | |
| --- | --- | --- | --- | --- | --- | --- | --- |
| **Variables** | **Coefficient** | **95% CI** | **p value** | **Variables** | **Coefficient** | **95% CI** | **p value** |
| Follow up(months) | 0.000 | -0.000 to 0 .001 | 0.493 | **-** | **-** | **-** | **-** |
| Age (months) | 0.000 | -0.000 to 0.001 | 0.436 | - | - | - | - |
| **Length of urethral stent indwelling(days)** | **0.006** | **0.000 to 0.011** | **0.036*** | Length of urethral stent indwelling(days) | 0.003 | -0.008 to 0.015 | 0.519 |
| Length of antibiotics usage(days) | 0.005 | -0.005 to 0.015 | 0.323 | - | - | - | - |
| **Length of penile dressing(days)** | **0.010** | **0.000 to 0.021** | **0.048*** | Length of penile dressing(days) | 0.006 | -0.009 to 0.022 | 0.383 |
| Percentage of chordee(%) | 0.019 | -0.135 to 0.175 | 0.774 |  |  |  |  |

*UCF* Urethrocutaneous fistula

* p<0.05
