# Supplementary material for: Complications Following Primary Repair of Non-proximal Hypospadias in Children: A Systematic Review and Meta-Analysis
Source: Front Pediatr. 2020 Dec 9;8:579364. doi: 10.3389/fped.2020.579364 (PMC7756017; doi:10.3389/fped.2020.579364)
Supplement: Supplemental Table 4 — Assessment of risks of bias of case series. [file Table_4.DOC]

Supplemental table 4 Assessment of risks of bias of case series

| **Author** | **Selection** | **Ascertainment** | | **Causality** | | | | **Reporting** |
| --- | --- | --- | --- | --- | --- | --- | --- | --- |
|  | **Does the patients represent the whole experience of the centers** | **Was the exposure adequately ascertained** | **Was the outcome adequately ascertained** | **Were other alternative causes that may explain the observation ruled out*** | **Was there a challenge/rechallenge phenomenon*** | **Was there a dose–response effect*** | **Was follow-up long enough for outcomes to occur** | **Is the cases described with sufficient to allow other investigators to replicate** |
| Orkiszewski,1990 | No | Yes | Yes | Yes | NA | NA | No | Yes |
| de Jong,1992 | Yes | Yes | Yes | Yes | NA | NA | Yes | Yes |
| Belloli,1994 | No | Yes | Yes | Yes | NA | NA | Yes | Yes |
| Retik,1994 | No | Yes | Yes | Yes | NA | NA | Yes | Yes |
| Meyer-Junghanel, 1995 | Yes | Yes | Yes | Yes | NA | NA | Yes | Yes |
| Keramidas,1995 | No | Yes | Yes | Yes | NA | NA | No | Yes |
| Van Horn, 1995 | Yes | Yes | Yes | Yes | NA | NA | Yes | Yes |
| Caione, 1997 | No | Yes | Yes | Yes | NA | NA | No | Yes |
| Jawad,1997 | No | Yes | Yes | Yes | NA | NA | Yes | Yes |
| Gray,2003 | Yes | Yes | Yes | Yes | NA | NA | Yes | Yes |
| Jayanthi,2003 | Yes | Yes | Yes | Yes | NA | NA | Yes | Yes |
| Elicevik,2004 | No | Yes | Yes | Yes | NA | NA | Yes | Yes |
| Nguyen,2004 | Yes | Yes | Yes | Yes | NA | NA | Yes | Yes |
| Stehr,2005 | No | Yes | Yes | Yes | NA | NA | Yes | Yes |
| Antao,2007 | No | Yes | Yes | Yes | NA | NA | Yes | Yes |
| Aslan,2007 | No | Yes | Yes | Yes | NA | NA | Yes | Yes |
| El-Kassaby,2008 | No | Yes | Yes | Yes | NA | NA | Yes | Yes |
| Akbiyik,2009 | No | Yes | Yes | Yes | NA | NA | Yes | Yes |
| Guarino,2009 | No | Yes | Yes | Yes | NA | NA | Yes | Yes |
| Abolyosr,2010 | No | Yes | Yes | Yes | NA | NA | Yes | Yes |
| Snodgrass,2010 | No | Yes | Yes | Yes | NA | NA | Yes | Yes |
| Yigiter,2010 | No | Yes | Yes | Yes | NA | NA | Yes | Yes |
| Bilici,2011 | No | Yes | Yes | Yes | NA | NA | Yes | Yes |
| El-Hawy,2013 | No | Yes | Yes | Yes | NA | NA | Yes | Yes |
| Yildiz,2013 | No | Yes | Yes | Yes | NA | NA | Yes | Yes |
| Esposito,2014 | No | Yes | Yes | Yes | NA | NA | No | Yes |
| Wishart,2014 | No | Yes | Yes | Yes | NA | NA | No | Yes |
| Bush,2016 | No | Yes | Yes | Yes | NA | NA | Yes | Yes |
| Moradi,2016 | No | Yes | Yes | Yes | NA | NA | Yes | Yes |
| Spinoit,2017 | Yes | Yes | Yes | Yes | NA | NA | Yes | Yes |
| Saavedra-Belaunde,2017 | No | Yes | Yes | Yes | NA | NA | Yes | Yes |
| Bagnara,2020 | No | Yes | Yes | Yes | NA | NA | Yes | Yes |

*Mostly relevant to cases of adverse drug events
