# Supplementary material for: Complications Following Primary Repair of Non-proximal Hypospadias in Children: A Systematic Review and Meta-Analysis
Source: Front Pediatr. 2020 Dec 9;8:579364. doi: 10.3389/fped.2020.579364 (PMC7756017; doi:10.3389/fped.2020.579364)
Supplement: Supplemental Table 5 — Assessment of risks of bias of comparative non-RCT studies. [file Table_5.DOC]

**Supplemental table 5** Assessment of risks of bias of comparative non-RCT studies

| **Studies** | **Domains** | | | | | | | **Overall judgement of risk of bias** |
| --- | --- | --- | --- | --- | --- | --- | --- | --- |
|  | **Bias due to confounding** | **Bias in selection of participants into the study** | **Bias in measurement of interventions** | **Bias due to departures from intended interventions** | **Bias due to missing data** | **Bias in measurement of outcomes** | **Bias in selection of the reported result** |  |
| Xu,2013 | Low | Moderate | Low | Moderate | Low | Low | Moderate | Moderate |
| Chalmers, 2015 | Low | Low | Low | Moderate | Moderate | Low | Moderate | Moderate |
| Buson,1994 | Moderate | Low | Low | Moderate | Moderate | Low | Moderate | Moderate |
| Hakim,1996 | Moderate | Moderate | Low | Moderate | Moderate | Low | Moderate | Moderate |
| Rampersad, 2017 | Moderate | Moderate | Low | Moderate | Low | Low | Moderate | Moderate |
| Samuel,2002 | Low | Low | Low | Moderate | Low | Low | Moderate | Moderate |
| Jia,2016 | Low | Moderate | Low | Moderate | Low | Low | Moderate | Moderate |
| Manuele，2019 | Moderate | Low | Low | Moderate | Moderate | Low | Moderate | Moderate |

*RCT* Randomized controlled trials
