# Supplementary material for: Complications Following Primary Repair of Non-proximal Hypospadias in Children: A Systematic Review and Meta-Analysis
Source: Front Pediatr. 2020 Dec 9;8:579364. doi: 10.3389/fped.2020.579364 (PMC7756017; doi:10.3389/fped.2020.579364)
Supplement: Supplemental Table 6 — Assessment of risks of bias of RCT. [file Table_6.DOC]

**Supplemental table 6** Assessment of risks of bias of RCT

| **Author** | **Random sequence generation** | **Allocation concealment** | **Blinding of participants and personnel** | **Incomplete outcome data** | **Selective reporting** | **Other sources of bias** | **Overall risk of bias** |
| --- | --- | --- | --- | --- | --- | --- | --- |
| Savanelli,2007 | High | Unclear | Unclear | Low | Low | Low | High |
| ElGanainy,2012 | High | Unclear | Unclear | Low | Low | Low | High |
| Mahmoud.2019 | Unclear | High | High | Low | Low | Low | High |
| Cimador,2013 | High | Unclear | Unclear | Low | Low | Low | High |
| Thomas,2015 | Unclear | High | Unclear | Low | Low | Low | High |
| Elbakry,2016 | Unclear | Unclear | Unclear | Low | Low | Low | Unclear |

*RCT* Randomized controlled trials
